# Transmission of rhinovirus in the Utah BIG-LoVE families: Consequences of age and household structure

**DOI:** 10.1371/journal.pone.0199388

**Published:** 2018-07-25

**Authors:** Frederick R. Adler, Chris Stockmann, Krow Ampofo, Andrew T. Pavia, Carrie L. Byington

**Affiliations:** 1 Department of Mathematics and Department of Biology, University of Utah, Salt Lake City, UT, United States of America; 2 Department of Pediatrics Medicine, University of Utah, Salt Lake City, UT, United States of America; 3 Health Sciences Center, Texas A&M University, College Station, TX, United States of America; University of Malaya, MALAYSIA

## Abstract

**Background:**

Common cold viruses create significant health and financial burdens, and understanding key loci of transmission would help focus control strategies. This study (1) examines factors that influence when individuals transition from a negative to positive test (acquisition) or a positive to negative test (loss) of rhinovirus (HRV) and other respiratory tract viruses in 26 households followed weekly for one year, (2) investigates evidence for intrahousehold and interhousehold transmission and the characteristics of individuals implicated in transmission, and (3) builds data-based simulation models to identify factors that most strongly affect patterns of prevalence.

**Methods:**

We detected HRV, coronavirus, paramyxovirus, influenza and bocavirus with the FilmArray polymerase chain reaction (PCR) platform (BioFire Diagnostics, LLC). We used logistic regression to find covariates affecting acquisition or loss of HRV including demographic characteristics of individuals, their household, their current infection status, and prevalence within their household and across the population. We apply generalized linear mixed models to test robustness of results.

**Results:**

Acquisition of HRV was less probable in older individuals and those infected with a coronavirus, and higher with a higher proportion of other household members infected. Loss of HRV is reduced with a higher proportion of other household members infected. Within households, only children and symptomatic individuals show evidence for transmission, while between households only a higher number of infected older children (ages 5-19) increases the probability of acquisition. Coronaviruses, paramyxoviruses and bocavirus also show evidence of intrahousehold transmission. Simulations show that age-dependent susceptibility and transmission have the largest effects on mean HRV prevalence.

**Conclusions:**

Children are most likely to acquire and most likely to transmit HRV both within and between households, with infectiousness concentrated in symptomatic children. Simulations predict that the spread of HRV and other respiratory tract viruses can be reduced but not eliminated by practices within the home.

## Introduction

The spread, prevalence and persistence of infectious diseases depends on the heterogeneity in the host population. This heterogeneity manifests as differences in host susceptibility, infectiousness, contact patterns, and duration of infection. Underlying causes of this epidemiological heterogeneity include basic host properties such as age, size and sex, dynamic properties such as health, immunity, and infection with other organisms, and social properties like household size or position within the contact network.

This study uses longitudinal data on a set of Utah households to detect host heterogeneity in susceptibility, transmission potential, and duration of rhinovirus and other respiratory tract infections. These infections are probably the most common symptomatic infections experienced by people in developed nations [1, 2], with human rhinovirus (HRV) generally the most common cause both in North America [1, 3, 4] and in the tropics and the southern hemisphere [5]. In this study, HRV is ubiquitous, infecting 93% of participants and 100% of households [1].

HRV and other viral respiratory infections cause significant economic and health burdens, and understanding their transmission pathways could help guide valuable control strategies [2, 6–8]. Many conditions are associated with HRV [8], including lower respiratory tract infections [7], asthma [9–12], chronic obstructive pulmonary disease (COPD) [11], exacerbations in cystic fibrosis [13–15], and morbidity in the elderly [8, 16]. Some studies have found HRV to be more deadly than influenza [17] in the elderly, and comparable to respiratory syncytial virus (RSV) in causing hospitalizations and wheezing in the very young [12, 18].

HRV does not infect the respiratory tract in isolation, and many studies have shown frequent coinfections with other viruses, with estimates ranging from 23.5% [19] to 66% over the course of illness [20], centered around 33% [5, 21]. How these viruses interact has been more difficult to establish, with one study finding a negative association of HRV with numerous other viruses, including bocavirus, coronaviruses, influenza A, and paramyxoviruses [19]. RSV may reduce the probability of HRV infection by 50%, although patients who are coinfected display symptoms of both viruses [22].

Numerous studies have documented patterns of HRV shedding. HRV tends to peak in the fall and spring [7], but is the dominant infection in summer [23]. Children have been implicated in numerous ways through higher prevalence in younger children [1, 4, 5, 24–26], in households with children [1, 26] and in parents [4]. The effects of sex have been less consistent, with studies showing higher prevalence in boys [24] or in women [26].

HRV can be detected and cause symptoms 10-11 hours after infection [27]. Asymptomatic infections are common, with as many as half of viral detections not associated with symptoms in the data presented in [1] and further analyzed here, with slightly lower figures in other studies [28, 29].

HRV shedding largely coincides with symptoms and transmission [30]. Asymptomatic infections generally have lower viral loads [28]. Mean shedding of HRV 10-11 days with the duration of symptoms correlated with the duration of shedding, and never exceeding 28 days [31]. Reinfection also plays an important role in HRV. Volunteer studies have shown that shedding ceases within 11-21 days, implying that longer infections are likely due to reinfection [32, 33]. Longitudinal studies of infants and children with HRV show frequent serotype changes in infections with long durations [34, 35].

There are two critical questions about HRV transmission: how it is transmitted, and by whom. Despite some inconsistent studies, the emerging consensus is that HRV transmission occurs both through contact and through aerosols [36, 37]. To quantify patterns of transmission among individuals, the majority of studies take place in households, quantifying both the extent of transmission, and the differences in susceptibility and infectiousness among household members. The simplest method tracks how many household members are infected after an identified index case [38–40] with studies generally finding higher transmission with children present [38, 40–42]. In the Seattle virus watch study, secondary transmission was higher when younger children introduced HRV into larger families [43]. Index case studies do have weaknesses, including being unable to effectively separate community from household transmission and in likely being biased toward more severe cases [44].

In the absence of an identified index case, other studies have looked for clusters of infection within families, finding increased antibodies to serotypes observed in other family members [45] or an infection in at least one other family member in nearly 70% of families [46].

Other statistical methods can overcome some of the weaknesses of these approaches. Maximum likelihood methods can be sensitive to assumptions about incubation period, and have low power to detect associations with age or sex when transmission rates are low [47]. To estimate the proportion of infections that occur within a household, models can enumerate all possible infection states to identify infections that from within the household [48]. An extension of this method used Markov Chain Monte Carlo to address the fact that chains of transmission are unobserved, and could separate the effects of antibody titer on susceptibility and of age on both susceptibility and infectiousness in influenza [44].

This study uses longitudinal data on 26 households tracked weekly over the course of one year to measure host heterogeneity in susceptibility, transmission potential, and duration of HRV infection. We begin with four main hypotheses based on earlier studies and the work of Peltola *et. al.* in particular [41].

1. Younger children will be both more likely to acquire and more likely to transmit HRV infections within and between households, We refer to increased likelihood of acquisition as higher susceptibility, although this cannot be distinguished from higher exposure with these data.
2. Larger households and households with younger children will have a higher prevalence of HRV,
3. Transmission will be lower from asymptomatic individuals,
4. HRV may have an antagonistic relationship with other respiratory tract viruses.

Our data analysis quantifies the role of heterogeneity in susceptibility and transmission, and provides estimates of the parameters needed for a simulation of HRV spread within and among households. We use this simulation to test the effects of inclusion or exclusion of different factors on patterns of prevalence. We focus on the extent to which removal or simplification of age-dependent susceptibility and transmission would be predicted to change the prevalence and distribution of HRV in simulated populations with different average household sizes.

## Methods

### Data

Data come from the Better Identification of Germs-Longitudinal Viral Epidemiology (BIG-LoVE) Study [1]. Briefly, this 52-week prospective investigation tracked 26 Utah households between August 2009 and August 2010. One household member collected weekly nasal samples and filled out an online symptom diary for each household member. Viral testing with the FilmArray RP multiplex respiratory virus panel (BioFire Diagnostics, Salt Lake City, Utah) was used to detect 17 respiratory tract infections, which we split into the five most common pathogen types:

- human rhinovirus (HRV)
- human bocavirus,
- coronaviruses (HKU1, NL63, OC43, and 229E),
- influenza A and B, and
- paramyxoviruses (human metapneumovirus, parainfluenza viruses 1-4, and respiratory syncytial virus).

We broke individuals into four age groups, determined at enrollment: 0-4, 5-17, 18-39, and 40-59 years. These represent preschool and school-aged children, and early and mid-adulthood.

For individuals with consecutive weekly nasal samples, we recorded acquisition of HRV for a given week if the test changed from negative in that week to positive in the next, and recorded NA (missing) for individuals who tested positive in the given week. We recorded loss of HRV if the test changed from positive in that week to negative in the next, and recorded NA for individuals who tested negative in the given week.

To characterize households, we found the youngest other household member (excluding the focal individual, but set to the age of that individual in households of size 1) and the household size. We computed the number of other household members testing positive for HRV, and found their frequency by dividing by the household size minus 1 (dividing by zero does not occur because there cannot be other infected individuals in households of size 1). To resolve transmission rates within households, we broke infected individuals up by age, sex, presence of symptoms, or combinations thereof. We term these the specific infection classes. For example, to define the intrahousehold specific infection class of people in the first age group, we found the number of infected individuals with age 0-4 in the household, excluding the focal individual.

We used our sample of 26 households to estimate interhousehold transmission. The households were not spatially clustered or otherwise connected, and we assume that the observed frequency and pattern of infection provide a representative sample of the entire community. When we find evidence of interhousehold transmission, we define specific age classes. For example, to define the interhousehold specific infection class of people in the second age group, we found the number of infected individuals with ages 5-17 outside the focal household.

### Regressions

We analyzed HRV acquisition and loss using logistic regression (the glm function with the binomial family in R [49]), which defaults to exclude missing data. Our initial set of covariates is given in Table 1. We include week^2^ to capture some nonlinear effects of season, and both age and age group, treated as an unordered factor, as candidate covariates to compare whether age in years adds predictive power over the coarser grouping. We applied univariate analysis to each covariate, and included all terms with *p* < 0.2 in a multivariate analysis. We used backward regression to remove all terms with *p* > 0.05 to arrive at a preliminary model and tested the resulting model in four ways: (1) forward regression by adding each other covariate one by one, (2) the Hosmer-Lemeshow test [50] for each covariate, (3) mixed effects models (glmmML function in R [51]) using household number as a random effect, (4) generalized additive models (gam function in R [52]) with a general function of week rather than a parametric form to test whether this absorbed any apparent signal of intrahousehold or interhousehold transmission.

**Table 1:** Initial set of covariates for logistic regression.

covariate	range
Week	1-52
Week^2^	1^2^ − 52^2^
Day length	9.68 − 14.56
Age (in years)	0—57
Age group	1—4
Sex	
Household size	1—8
Youngest	0—52
Health care worker	0 or 1
Day care	0 or 1
Full time student	0 or 1
Flu vaccine	0 or 1
Bocavirus positive	0 or 1
Coronavirus positive	0 or 1
Influenza positive	0 or 1
Parainfluenza positive	0 or 1
Number HRV positive in household	0—6
HRV frequency household	0.0—1.0
Number HRV positive outside household	2—40
Weeks of consecutive positive tests	0—7
Weeks of consecutive negative tests	0—48

When we found evidence of intrahousehold or interhousehold transmission or reinfection, we tested whether breaking up the number or fraction infected into specific infection classes based on age, presence of symptoms or sex improved the model fit. Models were compared with the AIC, and tested with mixed effect and generalized additive models as above. We did not correct for multiple comparisons because all models tested were submodels of a single multiple regression.

### Simulations

To examine the effects of age-dependent susceptibility, the structure of intrahousehold and interhousehold transmission, and of household size, we built a simulation model based on a simplified version of the final logistic regression model. This simplified model does not incorporate the effects of symptoms or interaction with other viruses which would require including their dynamics.

In the simplified model, the probability of acquisition of virus depends on four factors: week, age group, infections from within the household, and infections from outside the household. The probability of loss depends only on reinfections from within the household. We use logistic regression to find the logit for infection acquisition or loss, and convert to a probability with the inverse logit function.

We manipulate the simulation model in three ways to quantify their effects on patterns of HRV prevalence: 1) exclude specific components of observed susceptibility and transmission when building models to effectively absorb their effects into the intercept, 2) set coefficients of specific terms to zero after building the model, and 3) vary aspects of the population, such as household size.

- **Age group:** We either include or exclude the effects of age group on susceptibility when building the model of acquisition (termed age-dependent susceptibility or averaged susceptibility).
- **Intrahousehold transmission:** Models of intrahousehold transmission can include the overall fraction of infected household members (frequency-dependent intrahousehold transmission), that fraction broken into specific infection classes by age (age-dependent intrahousehold transmission), exclude intrahousehold transmission when building the models (averaged intrahousehold transmission), or set its coefficient to zero (zeroed-out intrahousehold transmission).
- **Interhousehold transmission:** Models of interhousehold transmission similarly break into frequency-dependent interhousehold transmission, age-dependent interhousehold transmission, averaged interhousehold transmission, and zeroed-out interhousehold transmission.
- **Loss of HRV:** We either include or exclude the effect of the number of infected household members when building the model of loss (termed density-dependent loss or averaged loss).

The full model with age-dependent susceptibility, specific infection classes for intrahousehold and interhousehold transmission, and reinfection within the household serves as a baseline. We vary these model components individually to create 64 models.

We ran simulations with two different household structures. First, we use the observed households in the BIG-LoVE study. Second, we create zero-truncated Poisson distributed household sizes [53] with different means, assuming at least one adult, a second individual with age group chosen at random based on census data, and any remaining individuals assumed to be children with age group chosen from census data (age groups 1-4 with probabilities 0.081452, 0.260122, 0.346232, 0.312194 respectively based on http://www.censusscope.org/us/chart_age.html).

Initial conditions are based on the probability of detection of HRV in the age groups in the first week of the study. The simulation computes the probability of acquisition or loss from the current state of each individual and the number of infections within or outside the household in that week. Simulations were run for 20 years, and replicated 3 times for each model. With Poisson-distributed household sizes, we choose 26 households with the Poisson parameter varied from 3 to 7, removing any households of size 0 and truncating to 10 any households with size greater than 10.

We use linear models (lm in R) to test the effects of model components and the population mean household size on overall mean prevalence and the slope of the relationship between household size and prevalence. For the latter, we find the slope by regressing mean prevalence within each household against household size.

## Results

Of the 5288 combinations of week and individual which could include data on HRV in both that and the next, the study includes data on 4107 transitions (77.7%). The number of transitions per individual ranges from 8-51, with a median of 42. Of the 733 positive HRV tests in the first 51 weeks of the study, we observed loss of HRV in 321 (43.8%), maintenance of HRV in 371 (50.6%), and no data in 41 (5.6%). Of the 3700 negative HRV tests during these weeks, we observe acquisition of HRV in 358 (9.7%), no acquisition in 3057 (82.6%), and no data in 285 (7.7%). In weeks with data to characterize HRV acquisition or loss, we observe 144 positive tests for coronavirus, 66 for paramyxovirus, 47 for influenza, and 188 for bocavirus.

**Fig 1:**
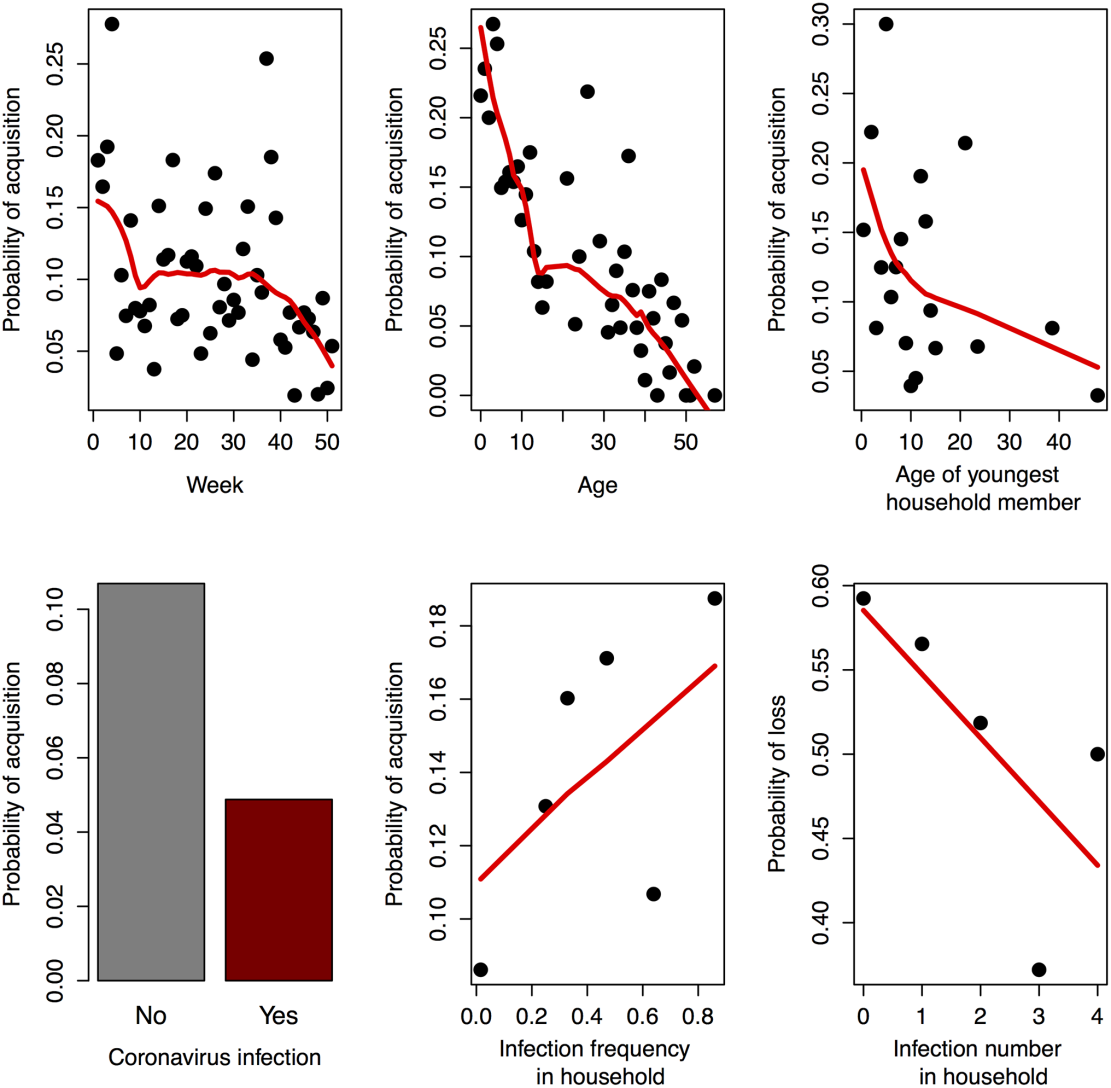
Univariate effects of covariates in the final generalized linear models. Probability of acquisition (a-e) or loss (f) of HRV as a function of a) week, b) age, c) age of youngest household member, d) concurrent positive test for coronavirus, e) fraction of other household members testing positive (pooled into six categories to smooth results), and f) number of other household members testing positive. Red lines showed a smoothed fit (supsmu function in R).

After model selection, five covariates were significant in predicting acquisition of HRV: week, age, youngest household member, infection with coronavirus, and frequency of HRV infection in other household members. The association with youngest household member is only marginally significant with a generalized linear mixed model (glmmML in R), but is included here because exclusion has little effect on the coefficients of the other covariates. Only a single covariate, number of HRV infections in other household members, predicts a reduced probability of loss of HRV (Table 2, Fig 1). We found no association with the number of consecutive weeks of positive tests on the probability of a transmission to a negative test, or of the number of consecutive weeks of negative tests on the probability of a transmission to a positive test.

**Table 2:** Logistic regression models for HRV acquisition and loss, with covariates defined in Table 1.

HRV acquisition			
Covariate	Odds Ratio	Confidence limits	p-value
Intercept	0.401	0.298-0.538	<0.001
Week	0.985	0.977-0.992	0.00014
Age	0.955	0.947-0.963	<0.0001
Youngest	0.976	0.958-0.993	0.0082
Coronavirus positive	0.333	0.128-0.712	0.011
HRV frequency in household	4.054	2.463-6.615	<0.0001
HRV loss			
Intercept	1.462	1.171-1.830	<0.0001
HRV positive in household	0.841	0.744-0.949	0.0052

**Fig 2:**
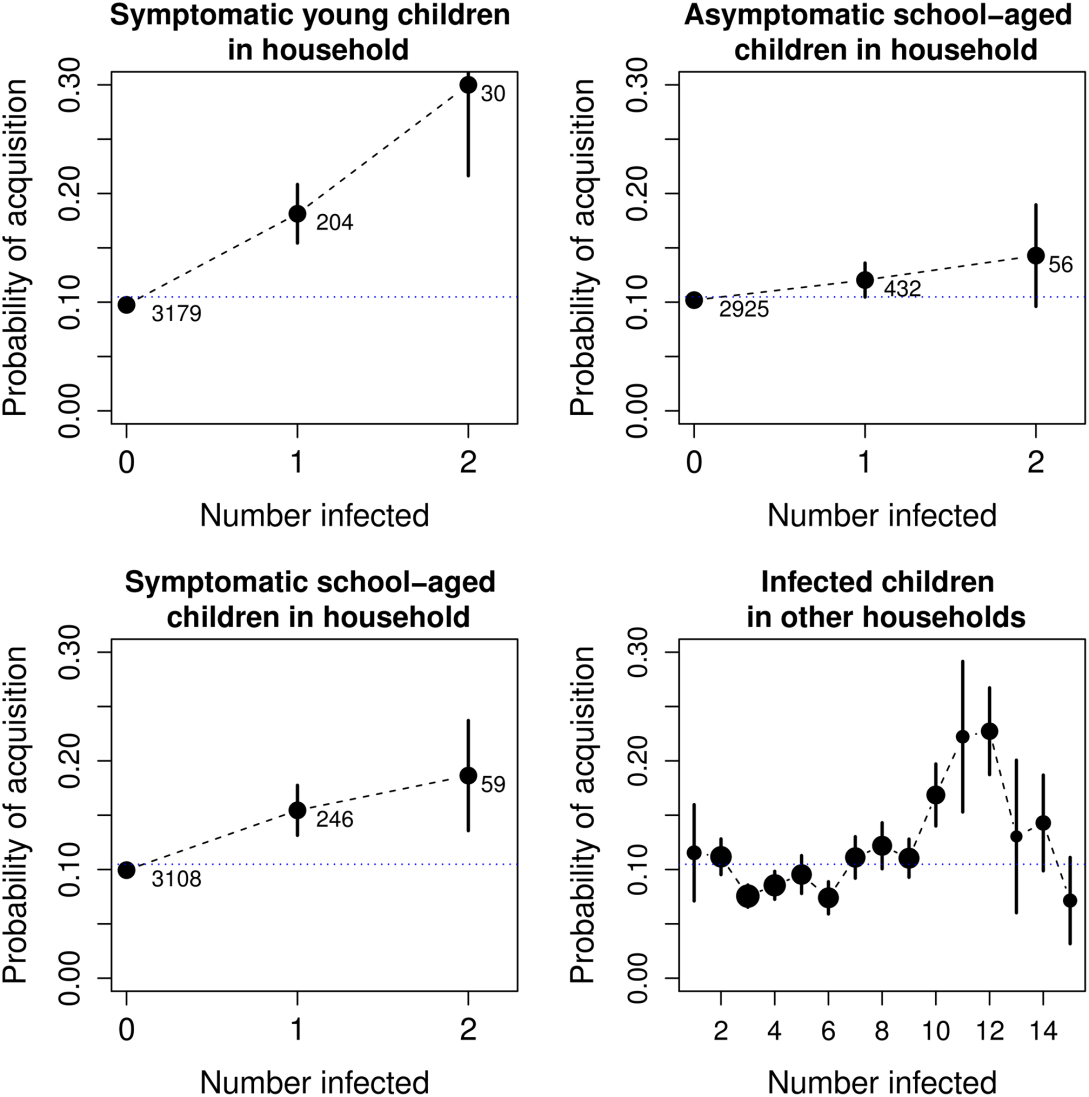
Univariate effects of significant specific infection classes. Average probability of infection as a function of specific infection classes. Number of data points indicated by numbers in a-c and by dot size in (d), ranging from the smallest value of 23 with 13 individuals infected to a largest value of 662 with 3 individuals infected. Error bars are one standard error.

For HRV acquisition, including specific infection classes by age and presence of symptoms within the household significantly improves the model fit, as does breaking individuals outside the household into specific infection classes by age (Fig 2). Sex has no association in any model, and no model found evidence for transmission by adults. Models with individuals aggregated by age (AIC 2086.7), presence of symptoms (AIC 2080.9), or age crossed by presence of symptoms (AIC 2077.4) improve the fit compared with the basic model (AIC 2094.6) (Table 3). For HRV loss, breaking into specific infection classes does not improve the model.

**Table 3:** Logistic regression models for HRV acquisition with specific infection classes. Within the household, all frequencies are the number of other individuals testing positive in the given class divided by household size minus 1. The population number is the number testing positive in the given class outside the household.

With specific infection classes based on age			
Covariate	Odds Ratio	Confidence limits	p-value
Intercept	0.265	0.171-0.408	<0.0001
Week	0.990	0.981-0.998	0.022
Age	0.953	0.945-0.961	<0.0001
Coronavirus positive	0.349	0.134-0.746	0.014
Youngest	0.980	0.962-0.997	0.032
Household HRV frequency in age group 1	8.761	3.686-20.26	<0.0001
Household HRV frequency in age group 2	4.426	1.853-10.28	0.00066
Population HRV number in age group 2	1.049	1.010-1.089	0.012
With specific infection classes based on presence of symptoms			
Intercept	0.285	0.179-0.454	<0.0001
Week	0.991	0.981-1.000	0.060
Age	0.954	0.946-0.962	<0.0001
Coronavirus positive	0.320	0.123-0.686	0.0082
Youngest	0.975	0.957-0.992	0.0054
Household HRV frequency with symptoms	8.978	4.567-17.38	<0.0001
Population HRV frequency with symptoms	1.042	1.004-1.082	0.030
With specific infection classes based on presence of symptoms and age			
Intercept	0.261	0.168-0.402	<0.0001
Week	0.991	0.982-0.9995	0.039
Age	0.952	0.944-0.960	<0.0001
Coronavirus positive	0.344	0.132-0.738	0.013
Youngest	0.978	0.959-0.995	0.016
Household HRV frequency in age group 2 without symptoms	3.325	1.024-10.08	0.039
Household HRV frequency in age group 1 with symptoms	21.86	7.889-58.54	<0.0001
Household HRV frequency in age group 2 with symptoms	9.963	2.861-32.13	0.00019
Population HRV number in age group 2	1.055	1.016-1.096	0.0056

### Regression results for other viruses

Although we observed fewer transitions to detect detailed patterns of transmission with viruses other than HRV, we found evidence for intrahousehold transmission for coronaviruses, paramyxoviruses, influenza, and bocavirus, and reduced susceptibility with older age in paramyxoviruses, influenza, and bocavirus. We detect a reduced rate of loss of coronaviruses in hosts infected with HRV and of bocavirus in male hosts, small households, and in households with a high frequency of concurrent infections (Table 4).

**Table 4:** Significant effects on acquisition and loss of other infections pooled into categories defined in the introduction. Results are from logistic regression (glm with the binomial family in R) after removing covariates that were not significant with a generalized linear mixed model using household number as a random effect. In each case, Frequency refers to the frequency of positive tests of the focal virus within the household. Models include quadratic terms to capture the non-linear effect of week, particularly for viruses with a stronger winter peak. The covariates chosen after model selection are the same when analyzed with generalized additive models (gam in R). No covariates significantly predict loss of paramyxovirus or influenza.

Covariate	Odds Ratio	Confidence limits	p-value
Coronavirus acquisition			
Intercept	0.00242	0.00077-0.0064	<0.0001
Week	1.322	1.202-1.475	<0.0001
Week^2^	0.993	0.991-0.996	<0.0001
Frequency	23.95	8.909-61.82	<0.0001
Coronavirus loss			
Intercept	2.237	1.538-3.314	<0.0001
HRV positive	0.224	0.0791-0.582	0.0029
Paramyxovirus acquisition			
Intercept	0.00257	0.000327- 0.0137	<0.0001
Age group	0.519	0.357-0.734	0.00034
Week	1.333	1.166-1.572	0.00014
Week^2^	0.994	0.991-0.997	0.00012
Frequency	32.82	3.431-208.03	0.00066
Influenza acquisition			
Intercept	0.00225	0.000240-0.0131	<0.0001
Week	3.669	2.126-7.285	<0.0001
Week^2^	0.906	0.861-0.943	<0.0001
Age	0.966	0.942-0.987	0.0027
Bocavirus acquisition			
Intercept	0.0982	0.0617-0.154	<0.0001
Age group	0.539	0.436-0.662	<0.0001
Frequency	73.66	32.79-164.48	<0.0001
Bocavirus loss			
Intercept	0.887	0.115-6.698	0.9068
Male sex	0.336	0.135-0.781	0.0139
Household size	1.870	1.285-2.840	0.0019
Frequency	0.0653	0.0207-0.187	<0.0001

### Simulation results

Simulations of HRV infection are based on regressions simplified in four ways: (1) we do not include the association with symptoms, which depend on infection status in a complex way, (2) we do not include the interaction with coronavirus which have their own dynamics, (3) we do not include the relatively small association with the youngest household member, and (4) we break age into age groups to provide easier comparison with census data.

**Fig 3:**
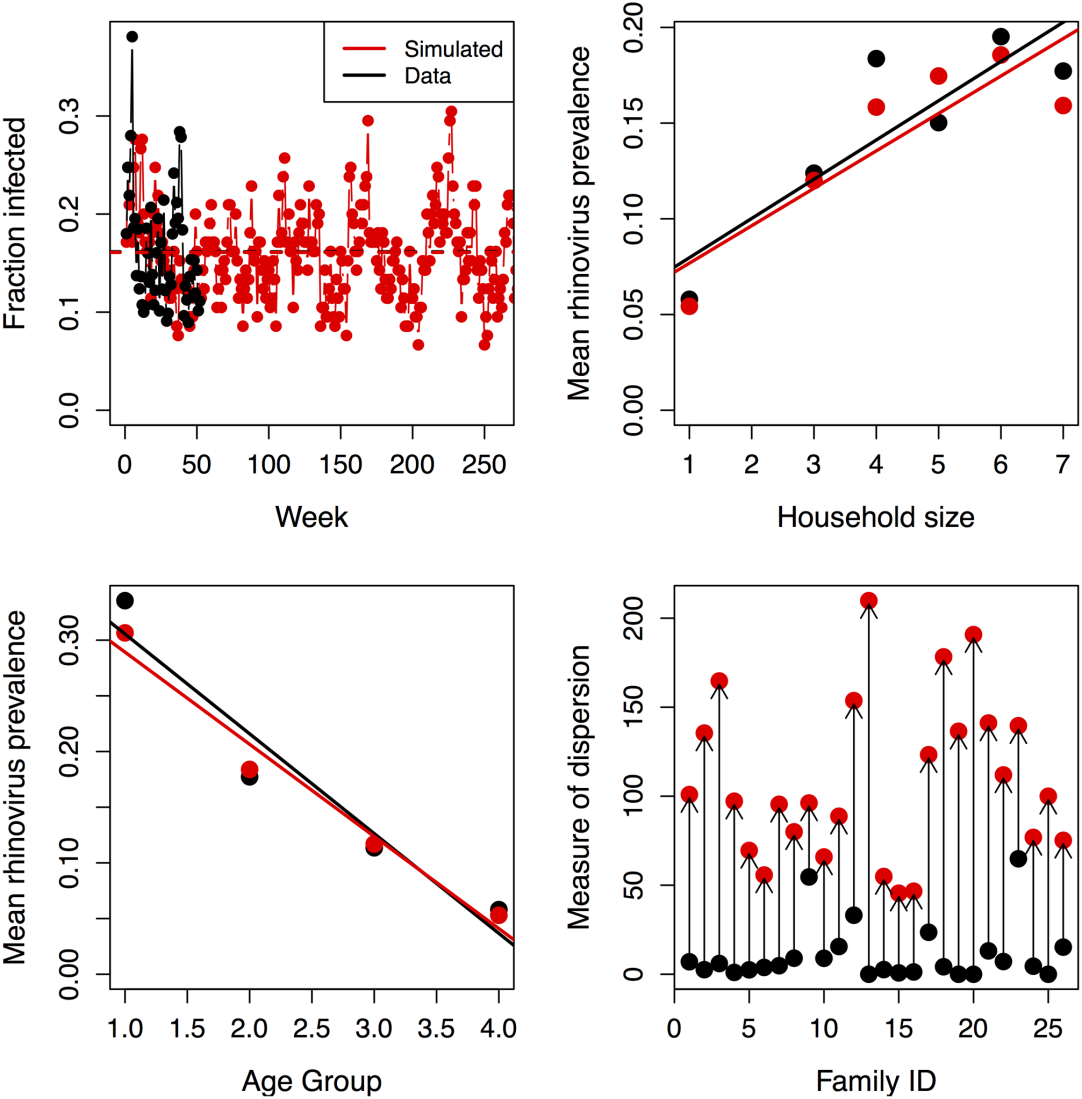
Results of simulations. We compare data (in black) with simulation (in red) for a) the trajectory for full year of data and first five years of the simulation, b) mean prevalence as a function of household size, c) mean prevalence as a function of age group, d) the index of dispersion *θ* for each household, with smaller values indicating a greater deviation from the binomial distribution (arrows connecting data to simulation added for clarity). The model includes age-dependent susceptibility, specific infection classes for age both within and between households and intrahousehold reinfection. Households match those at the beginning of the BIG-LoVE study. Coefficients of the logistic regression model are Intercept = -0.632, Week = -0.00943, Age group = -0.798, Household HRV frequency in age group 1 = 2.482, Household HRV frequency in age group 2 = 1.573, Population HRV number in age group 2 = 0.0495.

Using the ages and household sizes in the BIG-LoVE cohort, the simulation accurately reproduces HRV prevalence over time (Fig 3a), higher prevalence in larger households (Fig 3b), and lower prevalence with older hosts (Fig 3c). However, if we fit the number of individuals infected in each household with the beta-binomial distribution (package bbmle in R [54]), the values of the parameter *θ* in the simulation are consistently larger than those from the data. Larger values of *θ* produce a distribution closer to a binomial distribution where individuals have equal and independent probabilities of testing positive, indicating that the factors included in the simulation do not capture the full range of heterogeneity (Fig 3d).

We use the model to quantify sensitivity to four model components: age-dependent susceptibility, intrahousehold transmission, interhousehold transmission, and intrahousehold reinfection. Linear regressions quantified the effects of model components on overall mean prevalence and the slope of prevalence as a function of household size (Table 5).

**Table 5:** Simulated effects of model components on overall mean prevalence and on the slope of prevalence within households as a function of household size. The shorthand “intra” and “inter” refer to intrahousehold transmission and interhousehold transmission respectively.

Mean HRV prevalence in the population			
Experiment	Coefficient	Standard error	p-value
Averaged susceptibility	-0.0362	0.00538	<0.0001
Zeroed-out intra	-0.0408	0.00764	<0.0001
Frequency-dependent inter	-0.0470	0.00692	<0.0001
Averaged inter	-0.0525	0.00692	<0.0001
Zeroed-out inter	-0.0743	0.00692	<0.0001
Average household size	0.00941	0.00196	<0.0001
Slope of HRV prevalence with household size			
Averaged susceptibility	-0.0125	0.000609	<0.0001
Averaged intra	-0.00420	0.00128	0.00112
Zeroed-out intra	-0.00493	0.00128	0.00014
Zeroed-out inter	-0.00287	0.00131	0.029
Averaged loss	-0.00330	0.000910	0.00033
Average household size	-0.000954	0.000324	0.0035

Excluding the age-dependent susceptibility reduces population prevalence by 3.6% and setting intrahousehold transmission to zero reduces population prevalence by 4.0%. Simplifying age-dependent interhousehold transmission reduces population prevalence substantially (about 5% when the age-dependence is not included or when intrahousehold transmission is absorbed into the intercept), and setting the coefficient to zero reduces population prevalence by 7.4%. Increasing the average household size by one individual predicts an increase in population prevalence of nearly 1%.

**Fig 4:**
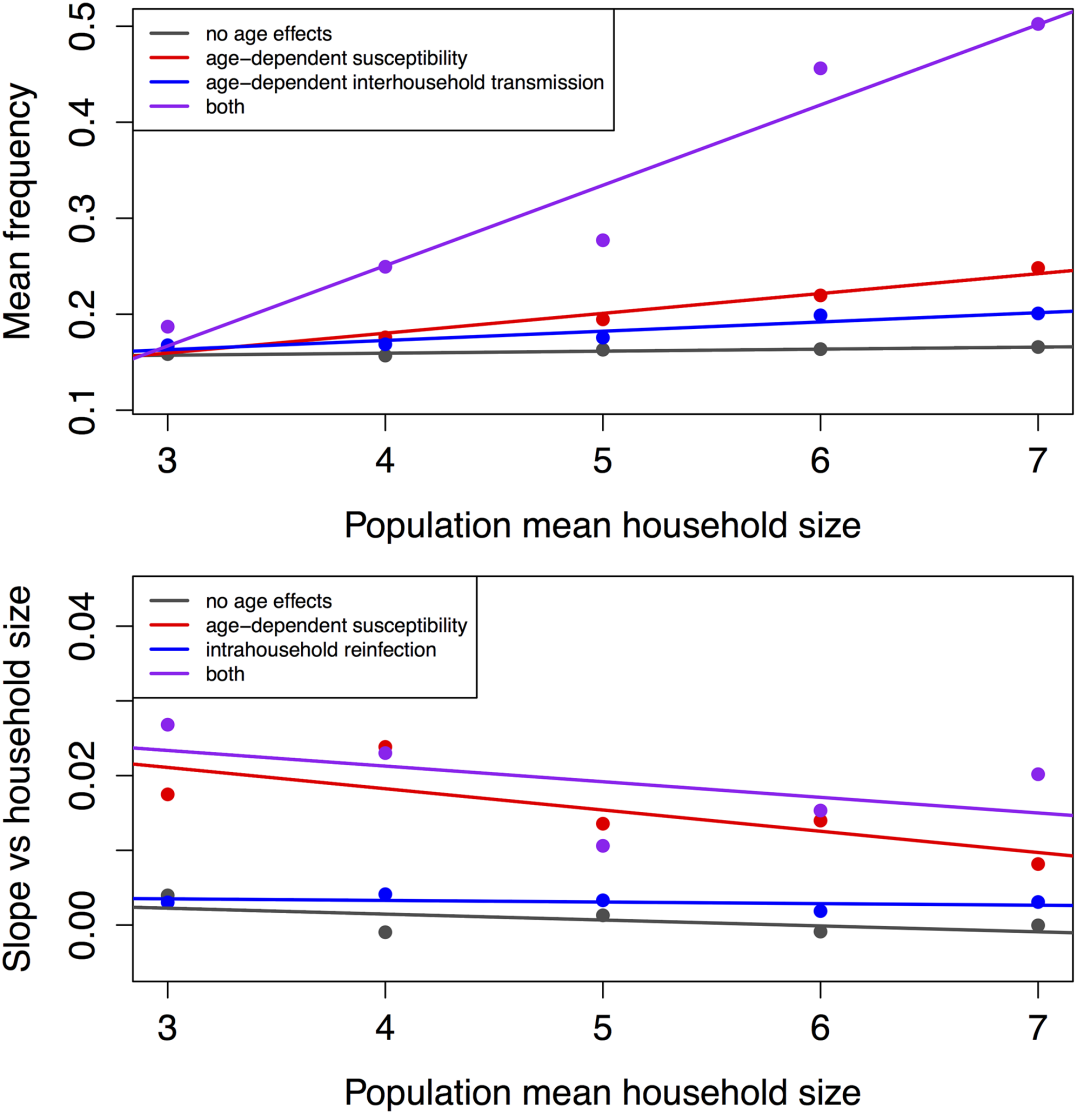
Effects of population mean household size and model structure on a) mean HRV prevalence in the entire population and b) the slope of HRV prevalence against household size within a population. The population mean household sizes follow the Poisson distribution with the given mean.

In a given simulation, prevalence in a household increases by 2% for each additional household member (slope of line in Fig 3b). This slope is reduced by 1.25% by neglecting age-dependent susceptibility, and by lesser amounts by simplifying intrahousehold transmission, zeroing out intrahousehold or interhousehold transmission, or by excluding reinfection (Fig 4b).

## Discussion

We used weekly samples and symptom diaries from 26 households in the Salt Lake City area to identify the factors that predict spread of HRV and other respiratory tract viruses within and between households. Our 733 HRV positive samples provide sufficient power to analyze how rates of acquisition and loss of viruses depend on the number and type of other infections in the household, and to fully parameterize a simulation model of this community.

Our main results replicate and quantify several previously identified and novel patterns of susceptibility, transmission, reinfection and virus interaction and cast new light on the four hypotheses we began with. 1. Children are indeed significantly more susceptible than adults, and more likely to transmit within households, and evidence for transmission between households emerges only in school-aged children. We find no evidence for transmission by adults. 2. Larger households and households with younger children thus have a higher prevalence of HRV, as predicted. 3. Transmission by individuals with symptoms is higher within households. 4. We find that testing positive for coronavirus reduces the probability of acquiring HRV but that loss of coronavirus is slower in individuals testing positive for HRV. In addition to these predictions, we found that loss of HRV is reduced when others in the household are infected, presumably due to reinfection, Finally, although we lack the power to definitively distinguish frequency-dependent from density-dependent transmission [55], intrahousehold transmission is better predicted by the frequency of infected individuals than by their number.

Although other viruses are more rare than HRV, providing substantially less power to detect mechanisms, we found evidence of intrahousehold transmission for coronaviruses, paramyxoviruses, influenza, and bocavirus, and reduced susceptibility with older age in paramyxoviruses, influenza, and bocavirus. We detect a reduced rate of loss of coronaviruses in hosts infected with HRV.

Simulation of HRV based on these data matches the broad structure of the data, both at the level of the population and as a function of age and household structure, although without capturing all forms of heterogeneity.

This study has numerous limitations. Weekly sampling is too coarse to capture the detailed transmission dynamics of a virus that is typically spread for only 10-11 days [31]. We cannot distinguish susceptibility from exposure with these methods. The study was small, and involved only 26 families over a single year and in a single region, making it difficult to generalize results or to correct for idiosyncratic differences among households. Our estimate of interhousehold transmission is based on treating the 26 households as a representative sample of the full population. Because the study is uncontrolled, so we cannot fully remove the effects of unmeasured covariates even with random effect models. Although compliance was generally high, there was sufficient missing data to further reduce power and potentially introduce reporting bias. Finally, the FilmArray has not been fully validated for HRV and the sensitivity and specificity could be lower than the ideal assumed here.

Because we did not distinguish serotypes, we can only infer that the reduced apparent loss rate of HRV infection is due to reinfection, and cannot directly trace pathways of transmission within the household. We have low power to detect adult transmission or transmission from any group with small numbers.

HRV is the most common cause of the common cold [1, 4] and is generally both mild and self-limiting [7]. However, HRV creates a substantial medical and economic burden [8]. It is comparable to RSV as a cause of hospitalization in infants [56] and comparable with influenza in causing morbidity in the elderly [17]. Severe early HRV increase risks of later asthma and can trigger asthma exacerbations [9]. Patients with COPD [11] or cystic fibrosis [14] face increased risks of exacerbations. There is some evidence that HRV has positive associations with *Streptococcus pneumoniae* and *Haemophilus influenzae* that cause bacterial pneumonia. The understanding of transmission provided here could help to protect key groups who are at high risk from those who are highly infectious.

These results open up several new lines of inquiry. Although HRV and coronaviruses have been discussed in parallel [2], the interactions detected here point towards an immunologically mediated mechanism that could reveal key aspects of susceptibility. The importance of symptoms in transmission motivates development of models that include a symptoms dynamics module, as in recent models of influenza [57], which could better target different infection stages for control.

Further studies are needed to validate these results. As those results become available, they can be embedded into a larger scale long-term model that includes aging, births, and other changes in household structure to investigate how predicted changes in demography could shape HRV transmission [58], and ideally include multiple viruses such as coronavirus. Studies that combine long-term tracking of individuals with detailed information on viral serotypes and immune status will enable us to parameterize a much more comprehensive set of models.
